# Overweight and Obesity Are Associated With Acute Kidney Injury and Acute Respiratory Distress Syndrome, but Not With Increased Mortality in Hospitalized COVID-19 Patients: A Retrospective Cohort Study

**DOI:** 10.3389/fendo.2021.747732

**Published:** 2021-12-14

**Authors:** Jamie van Son, Sabrina M. Oussaada, Aydin Şekercan, Martijn Beudel, Dave A. Dongelmans, Sander van Assen, Ingo A. Eland, Hazra S. Moeniralam, Tom P. J. Dormans, Colin A. J. van Kalkeren, Renée A. Douma, Daisy Rusch, Suat Simsek, Limmie Liu, Ruud S. Kootte, Caroline E. Wyers, Richard G. IJzerman, Joop P. van den Bergh, Coen D. A. Stehouwer, Max Nieuwdorp, Kasper W. ter Horst, Mireille J. Serlie

**Affiliations:** ^1^ Department of Endocrinology and Metabolism, Amsterdam Gastroenterology Endocrinology Metabolism, Amsterdam University Medical Centre (UMC), University of Amsterdam, Amsterdam, Netherlands; ^2^ Department of Surgery, Amsterdam University Medical Centre (UMC), University of Amsterdam, Amsterdam, Netherlands; ^3^ Department of Neurology, Amsterdam University Medical Centre (UMC), Amsterdam Neuroscience, University of Amsterdam, Amsterdam, Netherlands; ^4^ Department of Intensive Care, Amsterdam University Medical Centre (UMC), University of Amsterdam, Amsterdam, Netherlands; ^5^ Department of Internal Medicine/Infectious Diseases, Treant Zorggroep, Emmen, Netherlands; ^6^ Department of Internal Medicine, St. Antonius Hospital, Nieuwegein, Netherlands; ^7^ Department of Intensive Care, Zuyderland Medical Center, Heerlen, Netherlands; ^8^ Department of Internal Medicine, Flevo Hospital, Almere, Netherlands; ^9^ Department of Intensive Care Medicine, Martini Hospital, Groningen, Netherlands; ^10^ Department of Internal Medicine, Noordwest Ziekenhuisgroep, Alkmaar, Netherlands; ^11^ Department of Internal Medicine/Endocrinology, Amsterdam University Medical Centre (UMC), VU (Vrije Universiteit) University Medical Centre, Amsterdam, Netherlands; ^12^ Department of Internal Medicine and Cardiovascular Research Institute Maastricht (CARIM), Maastricht University Medical Centre, Maastricht, Netherlands; ^13^ Department of Acute Internal Medicine, Amsterdam University Medical Centre (UMC), University of Amsterdam, Amsterdam, Netherlands; ^14^ Department of Internal Medicine, Viecuri Medical Center, Noord-Limburg, Venlo, Netherlands; ^15^ Department of Internal Medicine, Amsterdam University Medical Centre (UMC), Diabetes Centre, Vrije Universiteit (VU) University Medical Centre, Amsterdam, Netherlands; ^16^ Department of Vascular Medicine, Amsterdam University Medical Centre (UMC), University of Amsterdam, Amsterdam, Netherlands

**Keywords:** COVID-19, obesity, overweight, complications, mortality

## Abstract

**Objective:**

To evaluate the association between overweight and obesity on the clinical course and outcomes in patients hospitalized with COVID-19.

**Design:**

Retrospective, observational cohort study.

**Methods:**

We performed a multicenter, retrospective, observational cohort study of hospitalized COVID-19 patients to evaluate the associations between overweight and obesity on the clinical course and outcomes.

**Results:**

Out of 1634 hospitalized COVID-19 patients, 473 (28.9%) had normal weight, 669 (40.9%) were overweight, and 492 (30.1%) were obese. Patients who were overweight or had obesity were younger, and there were more women in the obese group. Normal-weight patients more often had pre-existing conditions such as malignancy, or were organ recipients. During admission, patients who were overweight or had obesity had an increased probability of acute respiratory distress syndrome [OR 1.70 (1.26-2.30) and 1.40 (1.01-1.96)], respectively and acute kidney failure [OR 2.29 (1.28-3.76) and 1.92 (1.06-3.48)], respectively. Length of hospital stay was similar between groups. The overall in-hospital mortality rate was 27.7%, and multivariate logistic regression analyses showed that overweight and obesity were not associated with increased mortality compared to normal-weight patients.

**Conclusion:**

In this study, overweight and obesity were associated with acute respiratory distress syndrome and acute kidney injury, but not with in-hospital mortality nor length of hospital stay.

## Introduction

Severe acute respiratory syndrome coronavirus (SARS-CoV)-2 is a novel coronavirus that causes coronavirus disease 2019 (COVID-19). The virus first emerged in Wuhan (China) in December 2019 and rapidly spread worldwide (1). On March 11, 2020, COVID-19 was declared a pandemic by the World Health Organization. In most cases, the infection course is asymptomatic or mild, but symptoms progress in some patients, leading to respiratory failure and death (2, 3). Risk factors associated with disease severity and/or mortality include advanced age, male sex, socioeconomic deprivation, non-white ethnicity, and the presence of comorbidities such as hypertension, diabetes, and pre-existing respiratory or cardiovascular disease (4–9).

Obesity is defined as a body mass index (BMI) ≥ 30kg/m^2^ (10). It is a debilitating condition associated with a range of disorders such as type 2 diabetes, dyslipidemia, hypertension, cardiovascular disease, and cancer (11). Several of the cardiometabolic complications of obesity are risk factors for poor COVID-19 outcomes (12). Some reports also implicate obesity itself as a risk factor for severe COVID-19, with an increased risk of hospitalization, intensive care unit (ICU) admission, mechanical ventilation, and/or death (13–17). Several explanations have been proposed, including the presence of obesity-associated comorbidities, restrictive pulmonary function due to increased visceral and thoracic fat mass, low-grade systemic inflammation, and immune dysfunction (18–20). However, given the confounding links between obesity and other COVID-19 risk factors, it is currently unknown whether excess body weight is an independent risk factor for a poor COVID-19 outcome. Therefore, we evaluated the associations between overweight (defined as BMI 25-29.9 kg/m^2^) or obesity and the clinical course and outcomes in patients hospitalized with COVID-19 during the first wave in the Netherlands.

## Methods

### Study Design and Participants

This retrospective, multicenter, cohort study of patients with proven SARS-CoV-2 infection admitted to one of the participating medical centers in the Netherlands was part of the CovidPredict consortium (CovidPredict.org). Patients admitted during the first wave were included in the present analysis; in the Netherlands, the first wave was defined by the National Institute for Public Health and Environment (RIVM) as the period between February 27, 2020, and June 30, 2020 (21). Inclusion criteria were: i) a laboratory-confirmed SARS-CoV-2 infection using a PCR-based test, and ii) hospital admission during the defined period. Patients were excluded from the analyses if: i) BMI was unknown, not recorded, or <18.5 kg/m^2^), or ii) age < 18 years. The study protocol was reviewed by the medical ethics committees of the Amsterdam University Medical Centers (Amsterdam UMC; 20.131). Given the exceptional circumstances related to the COVID-19 crisis and in accordance with national guidelines and European privacy law, the need for informed consent was waived, and an opt-out procedure was communicated by press release. One of the participating centers (Maastricht University Medical Centre) did collect written informed consent from all their patients.

### Data Collection

Patient demographics, presenting symptoms, medical data, and the clinical course were extracted from electronic medical records using standardized electronic data collection forms. The patients height and weight were registered at the participating centers and the BMI was calculated by the authors with the formula: weight (kg)/[height (m)]^2^. Definitions that were used to record comorbid conditions are presented in **Supplemental Material S1**. All collected data were de-identified. In-hospital data were recorded from the moment of hospital admission until either hospital discharge or death. Clinical outcome data were recorded until December 10, 2020.

### Outcomes

The primary outcome was the association between overweight/obesity and mortality in hospitalized patients with COVID-19. Patients were categorized into normal weight (BMI 18.5-24.9 kg/m^2^), overweight (BMI 25–29.9 kg/m^2^) and obesity (BMI ≥30 kg/m^2^). Secondary outcomes were: i) COVID-19-related symptoms, ii) complications during admission, iii) length of hospital stay, iv) the need for and duration of mechanical ventilation, and v) admission to the ICU.

### Statistical Analyses

Categorical variables are expressed as counts and percentages (%). Continuous variables are expressed as mean (± standard deviation) or median [interquartile range] for parametric and nonparametric data, respectively. Categorical variables were compared using the χ2 or Fisher’s exact test and one-way ANOVA with Tukey’s test. Continuous variables were compared using the Kruskal-Wallis or Mann‐Whitney U tests, as appropriate. Binary outcome parameters were evaluated using logistic regression and are reported as odds ratio (OR) with a 95% confidence interval (CI). First, simple logistic regression was used to assess outcomes in patients who were overweight or had obesity compared with normal-weight patients. Next, multivariate regression was used to adjust results for age, sex, and the number of comorbidities.

All statistical analyses were performed using IBM SPSS Statistics v26.0 (SPSS, Inc., Chicago, Illinois). Results were considered significant if the *p*-value was < 0.05. Where appropriate, the Bonferroni method for multiple testing correction was used.

### Handling of Missing Data

We examined the data for selection bias due to missing BMI data. Missing data analyses were performed using the χ2 test for dichotomous (i.e., ‘sex’ and ‘whether or not the number of comorbidities was recorded’) and Little’s Missing Completely at Random (MCAR) test with the Mann-Whitney U test for continuous (i.e., ‘age’ and ‘length of hospital stay in days’) variables; BMI data were handled using pairwise deletion, whereas other variables were deleted list wise. An overview of the missing data analyses is presented in **Supplemental Materials S2**, **S3**.

## Results

### Participants

From February 27, 2020, until June 30, 2020, a total of 2597 COVID-19 patients were admitted to ten CovidPredict participating medical centers in the Netherlands. After excluding patients with missing BMI data (n=810), with underweight (n=25), or without positive PCR-based SARS-CoV-2 test (n=128), data from 1634 hospitalized patients with laboratory-proven COVID-19 were included in the present analysis. Their baseline characteristics and pre-existing conditions are presented in **Table 1**.

**Table 1:** Demographics and baseline characteristics of hospitalized patients with COVID-19.

	Normal weight (n = 473)	Overweight (n = 669)	Obese (n = 492)	*p*-value
**Male sex (%)**	301 (63.6)	471 (70.4)	268 (54.5)	<0.001
**Age (years)**	72.0 [60.0-79.1]	67.0 [58.0-77.0]	63.1 [53.1-74.0]	<0.001
**BMI (kg/m^2^)**	23.2 [21.8-24.2]	27.3 [26.2- 28.4]	33.1 [31.2-36.1]	<0.001
**Active smoker (%)**	18 (3.8)	26 (3.9)	20 (4.1)	0.977
**Alcohol abuse (%)**	10 (2.1)	24 (3.6)	8 (1.6)	0.075
**Comorbidities**				
**Hypertension (%)**	181 (38.3)	301 (45.0)	266 (54.1)	<0.001
**Diabetes, with complications (%)**	24 (5.1)	48 (7.2)	45 (9.1)	0.049
**Diabetes, without complications (%)**	72 (15.2)	110 (16.4)	121 (24.6)	<0.001
**Rheumatic disease (%)**	47 (9.9)	68 (10.2)	60 (12.2)	0.441
**Auto-immune disease (%)**	36 (7.6)	45 (6.7)	37 (7.5)	0.812
**Organ transplant**	13 (2.7)	8 (1.2)	4 (0.8)	0.033
**HIV and/or AIDS (%)**	1 (0.2)	2 (0.3)	5 (1)	0.208
**Chronic cardiac disease (%)**	158 (33.4)	206 (30.8)	140 (28.5)	0.250
**Asthma (%)**	37 (7.8)	71 (10.6)	65 (13.2)	0.025
**Other chronicpulmonary disease (%)**	87 (18.4)	107 (16.0)	103 (20.9)	0.097
**Chronic kidneydisease (%)**	52 (11.0)	63 (9.4)	46 (9.3)	0.614
**Mild liver disease (%)**	12 (2.5)	13 (1.9)	21 (4.3)	0.055
**Severe liver disease (%)**	5 (1.1)	8 (1.2)	5 (1.0)	0.953
**Chronic neurologicdisease (%)**	72 (15.2)	76 (11.4)	53 (10.8)	0.069
**Hematologic disease (%)**	30 (6.3)	29 (4.3)	13 (2.6)	0.020
**Dementia (%)**	18 (3.8)	21 (3.1)	8 (1.6)	0.112
**Malignancy (%)**	52 (11.0)	42 (6.3)	24 (4.9)	0.001

Comparison of demographic and characteristic data between patients with normal weight (BMI 18.5-24.9 kg/m^2^), overweight (BMI 25-29.9 kg/m^2^) and obesity BMI (≥30 kg/m^2^). Categorical data are count (%) and compared by χ2 or Fisher’s exact tests. Continuous data are median [IQR] and compared by Kruskal-Wallis test. Definitions of comorbidities are presented in **Supplemental Table S1**. An overall p-value <0.05 is considered statically significant. Post-hoc analysis between groups is presented in **Supplemental Table S4**.

Of 1634 included COVID-19 patients, 473 (28.9%) had normal weight (BMI 18.5-24.9 kg/m^2^), 669 (40.9%) had overweight (BMI 25-29.9 kg/m^2^), and 492 (30.1%) had obesity (BMI ≥ 30 kg/m^2^). Patients with overweight and obesity were younger than normal-weight patients, and more women were in the obese group. Patients with obesity more often suffered from hypertension, type 2 diabetes, or asthma, whereas the prevalence of transplant organ recipiency, hematological disease, and malignancies was lower in the obese group. The patients’ medication use registered at admission is presented in **Supplemental Table S5**.

### Presenting Symptoms and Findings

Reported symptoms at the time of hospital admission were mostly consistent with those previously reported for COVID-19 patients: the most frequently reported symptoms for all patients were fatigue/malaise (81.6%), fever (77.0%), and dyspnea (74.9%) (22). An overview of all reported COVID-19 symptoms in the patients with normal-weight, overweight, and obesity is presented in **Table 2**. Patients in the overweight and obese groups more often reported cough, dyspnea, headache, and chest pain.

**Table 2:** Symptoms at presentation.

	Normal weight	Overweight	Obese	*p*-value
**Fatigue/malaise**	343 (79.4)	512 (82.6)	379 (80.1)	0.367
**Fever**	321 (74.3)	471 (77.7)	363 (78.6)	0.270
**Dyspnea**	295 (66.1)	490 (76.9)	379 (80.1)	<0.001
**Coughing**	122 (30.0)	230 (39.2)	162 (37.2)	0.009
**Diarrhea**	124 (30.0)	176 (29.8)	161 (35.3)	0.123
**Nausea and/or vomiting**	93 (23.0)	157 (26.8)	121 (27.2)	0.311
**Myalgia**	74 (20.3)	127 (24.4)	105 (26.1)	0.156
**Headache**	61 (16.7)	120 (22.6)	114 (27.5)	0.001
**Chest pain**	55 (13.5)	118 (20.2)	102 (22.7)	0.002
**Abdominal pain**	54 (13.2)	84 (14.4)	74 (16.5)	0.391
**Rhinorrhea**	48 (13.4)	64 (12.6)	70 (17.0)	0.143
**Confusion**	70 (17.0)	76 (13.1)	52 (11.7)	0.071
**Sore throat**	35 (9.8)	52 (10.2)	51 (12.6)	0.391
**Anosmia**	21 (6.4)	32 (6.7)	35 (9.2)	0.273
**Hemoptysis**	12 (2.9)	24 (4.2)	18 (4.1)	0.571
**Arthralgia**	10 (2.8)	21 (4.1)	12 (3.0)	0.528
**Wheezing**	14 (3.6)	15 (2.8)	11 (2.6)	0.682

Difference in presenting symptoms during admission between patients with normal weight (BMI 18.5-24.9 kg/m^2^), overweight (BMI 25-29.9 kg/m^2^) and obesity BMI (≥ 30 kg/m^2^). Data are count (%) and compared by chi-square test or Fisher’s exact test. An overall p-value <0.05 is considered statically significant. Post-hoc analysis between groups is presented in **Supplemental Table S6**.

Of 1634 included COVID-19 patients, 473 (28.9%) had normal weight (BMI 18.5-24.9 kg/m^2^), 669 (40.9%) had overweight (BMI 25-29.9 kg/m^2^), and 492 (30.1%) had obesity (BMI ≥ 30 kg/m^2^). Patients with overweight and obesity were younger than normal-weight patients, and more women were in the obese group. Patients with obesity more often suffered from hypertension, type 2 diabetes, or asthma, whereas the prevalence of transplant organ recipiency, hematological disease, and malignancies was lower in the obese group. The patients’ medication use registered at admission is presented in **Supplemental Table S5**.

### Presenting Symptoms and Findings

Reported symptoms at the time of hospital admission were mostly consistent with those previously reported for COVID-19 patients: the most frequently reported symptoms for all patients were fatigue/malaise (81.6%), fever (77.0%), and dyspnea (74.9%) (22). An overview of all reported COVID-19 symptoms in the patients with normal-weight, overweight, and obesity is presented in **Table 2**. Patients in the overweight and obese groups more often reported cough, dyspnea, headache, and chest pain.

At admission, patients with obesity presented with higher plasma glucose concentrations than patients with normal-weight and overweight (7.3 [6.2-9.8], 6.8 [5.9-8.0], and 6.9 [6.1-8.6] mmol/L, respectively; p<0.001 and p=0.013, respectively). Lactate dehydrogenase was higher in patients who were overweight than the normal-weight group (356 (281-473) vs 329 [246-441] IU/L; p=0.038). C-reactive protein and creatinine concentrations did not differ between the groups (**Supplemental Table S7**).

### Complications During Hospital Admission

There was a high incidence of complications among the hospitalized patients with COVID-19: 290 (61.3%) patients with normal-weight, 430 (64.3%) overweight, and 278 (56.5%) obesity developed one or more complications (**Supplemental Table S8** **)**. Importantly, 11.3%, 13.5%, and 8.7% of patients in the normal-weight, overweight, and obese groups, respectively, had missing data on complications during hospital admission (**Supplemental Material S3c**). Nevertheless, patients who were overweight or had obesity did not appear to have an *overall* increased probability for any complication. There were some specific complications associated with overweight or obesity (**Table 3**). Most notably, patients who were overweight or had obesity had a higher probability of acute respiratory distress syndrome (ARDS) (OR 1.70 (1.26-2.30) and 1.40 (1.01-1.96), respectively) and acute kidney injury (AKI) (OR 2.29 (1.28-3.76) and 1.92 (1.06-3.48), respectively) during COVID-19 infection. There were no differences in vascular complications, including pulmonary embolism and deep venous thrombosis.

**Table 3:** Association of overweight and obesity with complications during admission.

	Overweight vs normal weight, unadjusted	Obesity vs normal weight, unadjusted	Overweight vs normal weight, adjusted	Obesity vs normal weight, adjusted
**Overall complications**	1.14 (0.89-1.45)	0.82 (0.63-1.06)	1.19 (0.93-1.52)	0.96 (0.73-1.25)
**Pulmonary complications**				
ARDS	1.73 (1.29-2.33)	1.22 (0.88-1.68)	1.70 (1.26-2.30)	1.40 (1.01-1.96)
Bacterial pneumonia	1.33 (0.90-1.95)	1.00 (0.65-1.53)	1.25 (0.85-1.84)	1.02 (0.66-1.58)
Aspergillosis pneumonia	1.07 (0.43-2.64)	1.26 (0.50-3.17)	*	*
Pneumothorax	0.8 (0.36-1.67)	0.35 (0.13-0.99)	*	*
**Cardiac complications**				
Congestive heart failure	0.45 (0.23-0.89)	0.84 (0.45-1.57)	0.50 (0.25-0.99)	1.13 (0.59-2.17)
Endocarditis, myocarditis or pericarditis	0.72 (0.18-2.89)	0.46 (0.08-2.54)	*	*
Cardiac arrhythmia	1.05 (0.71-1.57)	0.73 (0.47-1.15)	1.15 (0.77-1.73)	0.93 (0.58-1.49)
Cardiac ischemia	1.21 (0.43-3.36)	0.78 (0.24-2.56)	*	*
**Neurologic complications**				
Seizure	0.87 (0.26-2.86)	0.37 (0.07-1.92)	*	*
Cerebrovascular accident	1.37 (0.57-3.25)	1.05 (0.40-2.75)	*	*
**Coagulation disorders**				
Pulmonary embolism	1.25 (0.78-1.98)	0.89 (0.52-1.52)	1.18 (0.73-1.89)	1.01 (0.58-1.74)
Deep venous thrombosis	1.20 (0.60-2.41)	0.59 (0.24-1.42)	1.08 (0.54-2.18)	0.55 (0.22-1.35)
Disseminated intravascular coagulation	1.18 (0.28-4.96)	0.32 (0.03-3.08)	*	*
**Bacteremia**	1.41 (0.91-2.20)	1.15 (0.71-1.86)	1.31 (0.84-2.05)	1.21 (0.74-1.99)
**Anemia requiring transfusion**	1.03 (0.68-1.57)	0.71 (0.44-1.15)	1.02 (0.67-1.55)	0.70 (0.43-1.14)
**Rhabdomyolysis/myositis**	1.28 (0.53-3.07)	2.40 (1.04-5.50)	1.18 (0.49-2.84)	2.35 (1.01-5.47)
**Acute renal failure/injury requiring dialysis**	2.31 (1.35-3.94)	1.73 (0.97-3.10)	2.29 (1.28-3.76)	1.92 (1.06-3.48)
**Gastrointestinal hemorrhage**	2.70 (0.75-9.74)	1.88 (0.47-7.58)	*	*
**Liver failure**	0.91 (0.35-2.32)	0.58 (0.19-1.78)	*	*
**Delirium**	1.17 (0.85-1.62)	0.93 (0.65-1.33)	1.29 (0.92-1.79)	1.17 (0.81-1.69)

Multivariate logistic regression analyses comparing patients who are overweight (BMI 25-29.9 kg/m^2^) or have obesity (≥ 30 kg/m^2^) to normal weight patients (BMI 18.5-24.9 kg/m^2^). Adjusted analyses includes age, sex, and the number of comorbidities. Data are OR (95% CI).

^*^Analyses were not performed, because fewer than 40 events occurred.

### ICU Admission

Out of 1634 hospitalized patients with COVID-19, 467 (28.6%) patients were admitted to the ICU, most often for respiratory failure and the need for mechanical ventilation. Since patients with severe comorbidities may have had a non-ICU admission policy, we only provide descriptive data for this outcome. Within the normal-weight, overweight, and obese groups, 104 (22.0%), 223 (33.3%), and 140 (28.6%) patients, respectively, were admitted to the ICU.

### Length of Hospital and ICU Stay, and Days on Mechanical Ventilation

**Figure 1:**
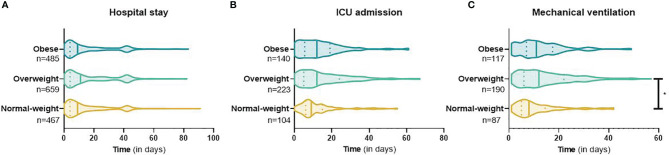
Length of hospital and ICU stay, and days on mechanical ventilation in hospitalized patients with COVID-19. Length of hospital **(A)** and ICU stay **(B)**, and days on mechanical ventilation **(C)** in COVID-19 patients with normal weight, overweight, and obesity. Data are presented as violin plots, with median (vertical line) and IQR (vertical dashes). *Post-hoc* Mann-Whitney-U test showed a significant difference in the duration of mechanical ventilation between the overweight and normal weight group, *p = <0.001. The table containing media [IQR] and statistical test result are presented in **Supplemental Table S7**.

Overall, patients with COVID-19 stayed a median [IQR] of 10 [4-26] days in the hospital. There were no differences in length of hospital stay between the normal-weight, overweight, and obese groups (**Figure 1A**). In addition, there were no group differences in the number of days in the ICU (**Figure 1B**). The number of days on a ventilator was higher in the overweight than in the normal weight group (**Figure 1C** and **Supplemental Table S9**). Adjustment for age, sex, and the number of pre-existing conditions did not alter these results.

### Clinical Outcomes: Destination After Discharge and Mortality

Of the 1414 patients for whom final outcome data were available, 1023 (72.3%) patients were discharged alive. There were no group differences in the destination after discharge (**Table 4**).

**Table 4:** Destination after discharge for COVID-19 patients with normal weight, overweight, or obesity who were discharged alive.

	Normal weight (n = 291)	Overweight (n = 408)	Obese (n = 324)
**Discharged to home**	231 (79.4%)	327 (80.1%)	258 (79.6%)
**Discharged to a nursing home**	12 (4.1%)	14 (3.4%)	14 (4.3%)
**Discharged to a rehabilitation unit**	44 (15.1%)	67 (16.4%)	52 (16.0%)
**Palliative discharge**	4 (1.4%)	0 (0.0%)	0 (0.0%)

Data are count (%).

A total of 391 (27.7%) patients died in the hospital: 124 (29.9%%), 166 (28.9%), and 101 (23.8%) in the normal-weight, overweight, and obese groups, respectively. Both univariate and multivariate logistic regression analyses demonstrated no difference in death amongst patients who were overweight or had obesity hospitalized with COVID-19, as compared to those with normal weight. Alternatively, using stepwise logistic regression, we observed that male sex [OR 1.65 (1.28–2.13)], age [OR 1.07 (1.06-1.09)], and the number of pre-existing conditions [OR 1.33 (1.24-1.43)] associated independently with death during hospital admission.

## Discussion

Data from this retrospective multicenter cohort study of hospitalized COVID-19 patients during the first wave of the pandemic in the Netherlands demonstrates that patients who are overweight or have obesity do not have an increased probability of in-hospital mortality nor an extended length of hospital stay as compared to normal-weight patients. However, both patients with overweight and obesity have an increased probability of developing ARDS and AKI during admission for COVID-19.

It is important to note that in this cohort the prevalence of overweight and obesity was 40.9% and 30.1%, respectively; while in the Dutch population in 2019 the prevalence of overweight and obesity amongst those aged 65-75 years (i.e., the age range in our cohort) was 42.5 and 17.7%, respectively (23). This indicates that patients with obesity were overrepresented in our cohort of hospitalized COVID-19 patients. This finding is in line with previous reports describing the increased probability of hospitalization for COVID-19 in patients with obesity (13, 24). Two possible explanations for this observation are that: i) obese individuals may be more prone to infection with SARS-CoV-2 (due to increased risk of exposure; time spent indoors; contact with healthcare professionals; immunological, or other factors), or ii) individuals with obesity and COVID-19 may be at higher risk of severe COVID-19 disease requiring hospital admission. Obese individuals have an altered immune system with chronic low-grade inflammation (25), and previous studies have implicated obesity to negatively impact immune function and response to viral infections and vaccines (26–28). Additionally, increased visceral adiposity has been linked to an elevated risk for adverse outcomes in septic ICU patients (29). Moreover, once admitted, the overweight and obese groups in our cohort had increased probability of ARDS and AKI; a finding that is consistent with previous reports (30). However, despite this, we found no increased probability of *overall* in-hospital complications or death in COVID-19 patients who are overweight or have obesity.

These observations are important for clinicians because they indicate that the presence of overweight or obesity per se should not influence important clinical decisions during in-hospital COVID-19 care, including decisions regarding ICU admission or the initiation of mechanical ventilation.

Our observation that overweight and obesity are not independent factors for in-hospital mortality may seem contrary to previous reports and popular media coverage (31–33), but several possible explanations are available. Firstly, obesity disproportionally impacts the lower socioeconomic classes (34, 35); in countries where income affects healthcare availability, this may confound COVID-19 outcomes in obese individuals (36), whereas both emergency care and the care for chronic conditions (including the cardiometabolic complications of obesity) are widely accessible, independent of socioeconomic status, in the Netherlands. Secondly, the median BMI of the obese group was 33.1 kg/m^2^, with only 44 patients classified as severely obese (i.e., BMI ≥ 40 kg/m^2^). In other cohorts, in-hospital mortality was increased in patients with severe obesity (BMI ≥ 35 kg/m^2^), but not in patients who had overweight or class 1 obesity (BMI 25-34.9 kg/m^2^) (33, 37, 38). A recent large multicenter study found that increased risk of death was limited to class 3 obesity (BMI ≥ 40 kg/m^2^) (39). Additionally, a meta-analysis confirmed this non-linear relationship between BMI and poor outcome in patients with COVID-19, in which the association became increasingly stronger from BMI 30-35 kg/m^2^ onwards (40). Thirdly, as described previously increased visceral adipose tissue is associated with adverse outcomes in ICU patients (29). More recent studies have corroborated this finding in patients with COVID-19 (41–43). In our cohort anthropomorphic data on body composition was not available. Measuring visceral adiposity with computed tomography scans to assess the risk for adverse outcomes in patients admitted with COVID-19 would not be feasible in the clinical practice. However, waist circumference and waist-to-hip ratio are simple and inexpensive methods to measure abdominal obesity and have proven to predict obesity-related health risks and mortality (44, 45). It would be interesting for future studies to add the measurement of waist circumference and/or waist-to-hip ratio as a supplement to BMI to predict adverse events and mortality in patients hospitalized with COVID-19. Lastly, patients who are overweight or have obesity may be at increased risk of severe COVID-19, requiring hospital admission, with similar outcomes once they are admitted; this would still make overweight/obesity a risk factor for COVID-19 related morbidity/mortality from a population point of view.

We found several group differences in specific complications during admission. Patients who were overweight or had obesity more frequently developed ARDS and, on average, patients who were overweight required longer mechanical ventilation. Notably, prolonged mechanical ventilation was not observed in patients with obesity. The data on mechanical ventilation shows a large interquartile range which could explain the need for a larger sample size to reach statistical significance. Two possible explanations for the increased ARDS probability in the overweight and obese group are: i) reduced lung volume and compliance due to the presence of adipose tissue around the rib cage, abdomen, and within the visceral cavities (46, 47), which impairs alveolar gas exchange (48, 49), and ii) increased airway hyperresponsiveness due to the chronic inflammatory state (50–53). It is important to note that even though patients with overweight and obesity more often developed ARDS they did not have a higher mortality rate. This observation has more frequently been described and is also referred to as ‘the obesity paradox’ (54). Several studies, including two large meta-analysis, have reported that obesity was associated with an increased risk of ARDS; however, lower risk of mortality (54–57). The underlying mechanisms of ‘ the obesity paradox’ remain unclear. A possible explanation could be an altered inflammatory response. Animal studies have shown that obese rodents with acute lung injury (ALI) had an anti-inflammatory phenotype which, after acute infection, could protect the lungs from further damage due to severe inflammation (58, 59). Additionally, lower circulating pro-inflammatory cytokines have been reported in humans with obesity and ALI (60). Next, patients who were overweight or had obesity also more often developed AKI requiring dialysis. Several factors such as age, diabetes, hypertension and baseline serum creatinine concentrations are associated with an increased risk for AKI in patients with COVID-19 (61). In this cohort, there was a higher prevalence of hypertension and diabetes in patients with overweight and obesity which could affect the renal function in these groups (62, 63). However, our analysis corrected for these factors and baseline creatinine concentrations did not differ between the groups. Nevertheless, obesity is known to be an independent risk factor for developing kidney disease (64, 65). The pathophysiology behind an increased risk of kidney disease in obesity is not completely understood, but factors such as peripheral insulin resistance, pro-inflammatory state, glomerular hyperfiltration, and dyslipidemia could increase the risk for AKI during illness (66–68). Moreover, in patients admitted to the ICU an increased BMI has previously been described to be an independent risk factor for developing AKI (69, 70). A finding which more recently has also been reported in patients with COVID-19 (71).

This retrospective study has several strengths and limitations. It describes a large cohort of hospitalized COVID-19 patients in the Netherlands, with relatively large normal-weight, overweight, and obese groups; it is a multicenter study, spanning both academic and general hospitals throughout the Netherlands; and the CovidPredict database records detailed individual patient data on pre-existing conditions, signs and symptoms at the time of hospital admission as well as events occurring during the hospital stay.

The first and most important limitation is that 46.3% of admitted patients in the original dataset had missing BMI data; given our primary outcome, these patients had to be excluded from all analyses. One may hypothesize that BMI is selectively registered in severely ill patients, thereby introducing bias. We performed several missing data analyses (**Supplemental Materials S2**, **S3**) to gain insight into the missing data. These analyses showed some minor and, in our opinion, clinically irrelevant differences between groups. Even though we do not think that these slight differences are likely to meaningfully impact our results on mortality outcome, a possible selection bias on registering BMI cannot be excluded. Secondly, we were unable to correct for socioeconomic status and ethnicity; factors associated with COVID-19 prevalence and mortality (72, 73). Thirdly, a small number of patients was discharged to another hospital (3.0%) after which they were lost to follow-up. Fourthly, during the first wave of the pandemic, there was no nationwide protocol on the treatment of COVID-19, which led to differences in treatment strategies between the participating hospitals. During the first wave in the Netherlands, the two most prescribed medications were hydroxychloroquine and remdesivir (74). More recent insights suggest that both have little-to-no effect on the need for mechanical ventilation, length of hospital stay, or overall mortality (75). Therefore, we do not believe that heterogeneity in therapeutic strategies introduced any bias. Fifthly, our data merely represents patients admitted to the hospital. Hence, we cannot exclude a bias introduced by hospital admission policies per se. Sixthly, patients with severe comorbidities may have had a non-ICU admission policy; introducing a bias in the main outcomes. Therefore, ICU admission rates were reported as descriptive data only. Finally, we stress that these data only apply to hospitalized patients with COVID-19.

In conclusion, during the first wave of the pandemic in the Netherlands, patients with obesity were overrepresented amongst hospitalized patients with COVID-19. Overweight and obesity were associated with the development of ARDS and AKI, but not with in-hospital mortality nor with the duration of hospital stay.

## Data Availability Statement

The datasets presented in this article are not readily available as they contain clinical information. Aggregated data can be obtained based upon reasonable request from the senior author.

## Ethics Statement

The study protocol was reviewed by the medical ethics committees of the Amsterdam University Medical Centers (Amsterdam UMC; 20.131). Given the exceptional circumstances related to the COVID-19 crisis and in accordance with national guidelines and European privacy law, the need for informed consent was waived, and an opt-out procedure was communicated by press release. One of the participating centers (Maastricht University Medical Centre) did collect written informed consent from all their patients.

## Author Contributions

MB organized the database. JS, SO, and MS contributed to the conception and design of the study. JS, SO, and AŞ performed the statistical analysis. JS and SO wrote the first draft of the manuscript. All authors contributed to manuscript revision, read and approved the submitted version.

## Funding

This work was supported by a grant from the Corona Research Foundation Amsterdam UMC.

## Conflict of Interest

The authors declare that the research was conducted in the absence of any commercial or financial relationships that could be construed as a potential conflict of interest.

## Publisher’s Note

All claims expressed in this article are solely those of the authors and do not necessarily represent those of their affiliated organizations, or those of the publisher, the editors and the reviewers. Any product that may be evaluated in this article, or claim that may be made by its manufacturer, is not guaranteed or endorsed by the publisher.

## References

[1] Zhu N Zhang D Wang W Li X Yang B Song J. A Novel Coronavirus From Patients With Pneumonia in China, 2019. N Engl J Med (2020) 382(8):727–33. doi: 10.1056/NEJMoa2001017 PMC7092803 31978945

[2] Kim GU Kim MJ Ra SH Lee J Bae S Jung J. Clinical Characteristics of Asymptomatic and Symptomatic Patients With Mild COVID-19. Clin Microbiol Infect (2020) 26(7):948 e1–3. doi: 10.1016/j.cmi.2020.04.040 PMC7252018 32360780

[3] Mizumoto K Kagaya K Zarebski A Chowell G. Estimating the Asymptomatic Proportion of Coronavirus Disease 2019 (COVID-19) Cases on Board the Diamond Princess Cruise Ship, Yokohama, Japan, 2020. Euro Surveill (2020) 25(10). doi: 10.2807/1560-7917.ES.2020.25.10.2000180 PMC7078829 32183930

[4] Yang J Zheng Y Gou X Pu K Chen Z Guo Q. Prevalence of Comorbidities and its Effects in Patients Infected With SARS-CoV-2: A Systematic Review and Meta-Analysis. Int J Infect Dis (2020) 94:91–5. doi: 10.1016/j.ijid.2020.03.017 PMC7194638 32173574

[5] Wu Z McGoogan JM. Characteristics of and Important Lessons From the Coronavirus Disease 2019 (COVID-19) Outbreak in China: Summary of a Report of 72314 Cases From the Chinese Center for Disease Control and Prevention. JAMA (2020) 323(13):1239–42. doi: 10.1001/jama.2020.2648 32091533

[6] Nandy K Salunke A Pathak SK Pandey A Doctor C Puj K. Coronavirus Disease (COVID-19): A Systematic Review and Meta-Analysis to Evaluate the Impact of Various Comorbidities on Serious Events. Diabetes Metab Syndr (2020) 14(5):1017–25. doi: 10.1016/j.dsx.2020.06.064 PMC7331565 32634716

[7] Grasselli G Zangrillo A Zanella A Antonelli M Cabrini L Castelli A. Baseline Characteristics and Outcomes of 1591 Patients Infected With SARS-CoV-2 Admitted to ICUs of the Lombardy Region, Italy. JAMA (2020) 323(16):1574–81. doi: 10.1001/jama.2020.4031 PMC7136855 32250385

[8] Peckham H de Gruijter NM Raine C Radziszewska A Ciurtin C Wedderburn LR. Male Sex Identified by Global COVID-19 Meta-Analysis as a Risk Factor for Death and ITU Admission. Nat Commun (2020) 11(1):6317. doi: 10.1038/s41467-020-19741-6 33298944 PMC7726563

[9] Holman N Knighton P Kar P O'Keefe J Curley M Weaver A. Risk Factors for COVID-19-Related Mortality in People With Type 1 and Type 2 Diabetes in England: A Population-Based Cohort Study. Lancet Diabetes Endocrinol (2020) 8(10):823–33. doi: 10.1016/S2213-8587(20)30271-0 PMC7426091 32798471

[10] Fact Sheet Obesity and Overweight 2016 03-01-2016. Available at: http://www.who.int/mediacentre/factsheets/fs311/en/.

[11] Grundy SM. Metabolic Complications of Obesity. Endocrine (2000) 13(2):155–65. doi: 10.1385/ENDO:13:2:155 11186217

[12] Collaborators, G.B.D.O Afshin A Forouzanfar MH Reitsma MB Sur P Estep K. Health Effects of Overweight and Obesity in 195 Countries Over 25 Years. N Engl J Med (2017) 377(1):13–27. doi: 10.1056/NEJMoa1614362 28604169 PMC5477817

[13] Popkin BM Du S Green WD Beck MA Algaith T Herbst CH. Individuals With Obesity and COVID-19: A Global Perspective on the Epidemiology and Biological Relationships. Obes Rev (2020) 21(11):e13128. doi: 10.1111/obr.13128 32845580 PMC7461480

[14] Lighter J Phillips M Hochman S Sterling S Johnson D Francois F. Obesity in Patients Younger Than 60 Years Is a Risk Factor for COVID-19 Hospital Admission. Clin Infect Dis (2020) 71(15):896–7. doi: 10.1093/cid/ciaa415 PMC7184372 32271368

[15] Hamer M Kivimaki M Gale CR Batty GD. Lifestyle Risk Factors, Inflammatory Mechanisms, and COVID-19 Hospitalization: A Community-Based Cohort Study of 387,109 Adults in UK. Brain Behav Immun (2020) 87:184–7. doi: 10.1016/j.bbi.2020.05.059 PMC7245300 32454138

[16] Zhou Y Yang Q Chi J Dong B Lv W Shen L. Comorbidities and the Risk of Severe or Fatal Outcomes Associated With Coronavirus Disease 2019: A Systematic Review and Meta-Analysis. Int J Infect Dis (2020) 99:47–56. doi: 10.1016/j.ijid.2020.07.029 32721533 PMC7381888

[17] Simonnet A Chetboun M Poissy J Raverdy V Noulette J Duhamel A. High Prevalence of Obesity in Severe Acute Respiratory Syndrome Coronavirus-2 (SARS-CoV-2) Requiring Invasive Mechanical Ventilation. Obesity (Silver Spring) (2020) 28(7):1195–9. doi: 10.1002/oby.23006 PMC7262326 32271993

[18] Petersen A Bressem K Albrecht J Thiess HM Vahldiek J Hamm B. The Role of Visceral Adiposity in the Severity of COVID-19: Highlights From a Unicenter Cross-Sectional Pilot Study in Germany. Metabolism (2020) 110:154317. doi: 10.1016/j.metabol.2020.154317 32673651 PMC7358176

[19] Alwarawrah Y Kiernan K MacIver NJ. Changes in Nutritional Status Impact Immune Cell Metabolism and Function. Front Immunol (2018) 9:1055. doi: 10.3389/fimmu.2018.01055 29868016 PMC5968375

[20] Huttunen R Syrjanen J. Obesity and the Risk and Outcome of Infection. Int J Obes (Lond) (2013) 37(3):333–40. doi: 10.1038/ijo.2012.62 22546772

[21] National Institute for Public Health and Environment R.O.t. Het Nieuwe Coronavirus in Nederland. And W.i.h.v.t.d.e.g.e.d.t.g.R.f (2020). Available at: https://www.rivm.nl/documenten/verschil-tussen-eerste-en-tweede-golf-corona.

[22] Grant MC Geoghegan L Arbyn M Mohammed Z McGuinness L Clarke EL. The Prevalence of Symptoms in 24,410 Adults Infected by the Novel Coronavirus (SARS-CoV-2; COVID-19): A Systematic Review and Meta-Analysis of 148 Studies From 9 Countries. PloS One (2020) 15(6):e0234765. doi: 10.1371/journal.pone.0234765 32574165 PMC7310678

[23] StatLine: Lengte En Gewicht Van Personen, Ondergewicht En Overgewicht; Vanaf 1981.

[24] Toussie D Voutsinas N Finkelstein M Cedillo MA Manna S Maron SZ. Clinical and Chest Radiography Features Determine Patient Outcomes in Young and Middle-Aged Adults With COVID-19. Radiology (2020) 297(1):E197–206. doi: 10.1148/radiol.2020201754 PMC7507999 32407255

[25] Gregor MF Hotamisligil GS. Inflammatory Mechanisms in Obesity. Annu Rev Immunol (2011) 29:415–45. doi: 10.1146/annurev-immunol-031210-101322 21219177

[26] Karlsson EA Sheridan PA Beck MA. Diet-Induced Obesity Impairs the T Cell Memory Response to Influenza Virus Infection. J Immunol (2010) 184(6):3127–33. doi: 10.4049/jimmunol.0903220 20173021

[27] Louie JK Acosta M Samuel MC Schechter R Vugia DJ Harriman K. A Novel Risk Factor for a Novel Virus: Obesity and 2009 Pandemic Influenza A (H1n1). Clin Infect Dis (2011) 52(3):301–12. doi: 10.1093/cid/ciq152 21208911

[28] Sheridan PA Paich HA Handy J Karlsson EA Hudgens MG Sammon AB. Obesity is Associated With Impaired Immune Response to Influenza Vaccination in Humans. Int J Obes (Lond) (2012) 36(8):1072–7. doi: 10.1038/ijo.2011.208 PMC3270113 22024641

[29] Pisitsak C Lee JG Boyd JH Coxson HO Russell JA Walley KR. Increased Ratio of Visceral to Subcutaneous Adipose Tissue in Septic Patients Is Associated With Adverse Outcome. Crit Care Med (2016) 44(11):1966–73. doi: 10.1097/CCM.0000000000001870 27513541

[30] Huang Y Lu Y Huang YM Wang M Ling W Sui Y. Obesity in Patients With COVID-19: A Systematic Review and Meta-Analysis. Metabolism (2020) 113:154378. doi: 10.1016/j.metabol.2020.154378 33002478 PMC7521361

[31] Steinberg E Wright E Kushner B. In Young Adults With COVID-19, Obesity Is Associated With Adverse Outcomes. West J Emerg Med (2020) 21(4):752–5. doi: 10.5811/westjem.2020.5.47972 PMC7390557 32726235

[32] Giacomelli A Ridolfo AL Milazzo L Oreni L Bernacchia D Siano M. 30-Day Mortality in Patients Hospitalized With COVID-19 During the First Wave of the Italian Epidemic: A Prospective Cohort Study. Pharmacol Res (2020) 158:104931. doi: 10.1016/j.phrs.2020.104931 32446978 PMC7242199

[33] Palaiodimos L Kokkinidis DG Li W Karamanis D Ognibene J Arora S. Severe Obesity, Increasing Age and Male Sex are Independently Associated With Worse in-Hospital Outcomes, and Higher in-Hospital Mortality, in a Cohort of Patients With COVID-19 in the Bronx, New York. Metabolism (2020) 108:154262. doi: 10.1016/j.metabol.2020.154262 32422233 PMC7228874

[34] Pavela G Lewis DW Locher J Allison DB. Socioeconomic Status, Risk of Obesity, and the Importance of Albert J. Stunkard. Curr Obes Rep (2016) 5(1):132–9. doi: 10.1007/s13679-015-0185-4 PMC4798886 26746415

[35] Pigeyre M Rousseaux J Trouiller P Dumont J Goumidi L Bonte D. How Obesity Relates to Socio-Economic Status: Identification of Eating Behavior Mediators. Int J Obes (Lond) (2016) 40(11):1794–801. doi: 10.1038/ijo.2016.109 27377952

[36] Bann D Johnson W Li L Kuh D Hardy R. Socioeconomic Inequalities in Body Mass Index Across Adulthood: Coordinated Analyses of Individual Participant Data From Three British Birth Cohort Studies Initiated in 1946, 1958 and 1970. PloS Med (2017) 14(1):e1002214. doi: 10.1371/journal.pmed.1002214 28072856 PMC5224787

[37] Tartof SY Qian L Hong V Wei R Nadjafi RF Fischer H. Obesity and Mortality Among Patients Diagnosed With COVID-19: Results From an Integrated Health Care Organization. Ann Intern Med (2020) 173(10):773–81. doi: 10.7326/M20-3742 PMC7429998 32783686

[38] Goyal P Ringel JB Rajan M Choi JJ Pinheiro LC Li HA. Obesity and COVID-19 in New York City: A Retrospective Cohort Study. Ann Intern Med (2020) 173(10):855–8. doi: 10.7326/M20-2730 PMC7384267 32628537

[39] Hendren NS de Lemos JA Ayers C Das SR Rao A Carter S. Association of Body Mass Index and Age With Morbidity and Mortality in Patients Hospitalized With COVID-19: Results From the American Heart Association COVID-19 Cardiovascular Disease Registry. Circulation (2020) 143(2):135–44. doi: 10.1161/CIRCULATIONAHA.121.054556 33200947

[40] Pranata R Lim MA Yonas E Vania R Lukito AA Siswanto BB. Body Mass Index and Outcome in Patients With COVID-19: A Dose-Response Meta-Analysis. Diabetes Metab (2021) 47(2):101178. doi: 10.1016/j.diabet.2020.07.005 32738402 PMC7388778

[41] Battisti S Pedone C Napoli N Russo E Agnoletti V Nigra SG. Computed Tomography Highlights Increased Visceral Adiposity Associated With Critical Illness in COVID-19. Diabetes Care (2020) 43(10):e129–30. doi: 10.2337/dc20-1333 32753457

[42] Goehler A Hsu TH Seiglie JA Siedner MJ Lo J Triant V. Visceral Adiposity and Severe COVID-19 Disease: Application of an Artificial Intelligence Algorithm to Improve Clinical Risk Prediction. Open Forum Infect Dis (2021) 8(7):ofab275. doi: 10.1093/ofid/ofab275 34258315 PMC8244656

[43] Yang Y Ding L Zou X Shen Y Hu D Hu X. Visceral Adiposity and High Intramuscular Fat Deposition Independently Predict Critical Illness in Patients With SARS-CoV-2. Obesity (Silver Spring) (2020) 28(11):2040–8. doi: 10.1002/oby.22971 PMC7405187 32677752

[44] Janssen I Katzmarzyk PT Ross R. Waist Circumference and Not Body Mass Index Explains Obesity-Related Health Risk. Am J Clin Nutr (2004) 79(3):379–84. doi: 10.1093/ajcn/79.3.379 14985210

[45] Jayedi A Soltani S Zargar MS Khan TA Shab-Bidar S. Central Fatness and Risk of All Cause Mortality: Systematic Review and Dose-Response Meta-Analysis of 72 Prospective Cohort Studies. BMJ (2020) 370:m3324. doi: 10.1136/bmj.m3324 32967840 PMC7509947

[46] Ladosky W Botelho MA Albuquerque JP Jr. Chest Mechanics in Morbidly Obese non-Hypoventilated Patients. Respir Med (2001) 95(4):281–6. doi: 10.1053/rmed.2001.1035 11316110

[47] Kress JP Pohlman AS Alverdy J Hall JB. The Impact of Morbid Obesity on Oxygen Cost of Breathing (VO(2RESP)) at Rest. Am J Respir Crit Care Med (1999) 160(3):883–6. doi: 10.1164/ajrccm.160.3.9902058 10471613

[48] Holley HS Milic-Emili J Becklake MR Bates DV. Regional Distribution of Pulmonary Ventilation and Perfusion in Obesity. J Clin Invest (1967) 46(4):475–81. doi: 10.1172/JCI105549 PMC442031 6021200

[49] Naimark A Cherniack RM. Compliance of the Respiratory System and its Components in Health and Obesity. J Appl Physiol (1960) 15:377–82. doi: 10.1152/jappl.1960.15.3.377 14425845

[50] Chinn S Jarvis D Burney P. Relation of Bronchial Responsiveness to Body Mass Index in the ECRHS. European Community Respiratory Health Survey. Thorax (2002) 57(12):1028–33. doi: 10.1136/thorax.57.12.1028 PMC1758811 12454296

[51] Jang AS Lee JH Park SW Shin MY Kim DJ Park CS. Severe Airway Hyperresponsiveness in School-Aged Boys With a High Body Mass Index. Korean J Intern Med (2006) 21(1):10–4. doi: 10.3904/kjim.2006.21.1.10 PMC3891057 16646558

[52] Kaplan TA Montana E. Exercise-Induced Bronchospasm in Nonasthmatic Obese Children. Clin Pediatr (Phila) (1993) 32(4):220–5. doi: 10.1177/000992289303200407 8462234

[53] Gokbel H Atas S. Exercise-Induced Bronchospasm in Nonasthmatic Obese and Nonobese Boys. J Sports Med Phys Fitness (1999) 39(4):361–4. 10726439

[54] Zhi G Xin W Ying W Guohong X Shuying L. "Obesity Paradox" in Acute Respiratory Distress Syndrome: Asystematic Review and Meta-Analysis. PloS One (2016) 11(9):e0163677. doi: 10.1371/journal.pone.0163677 27684705 PMC5042414

[55] Gong MN Bajwa EK Thompson BT Christiani DC. Body Mass Index is Associated With the Development of Acute Respiratory Distress Syndrome. Thorax (2010) 65(1):44–50. doi: 10.1136/thx.2009.117572 19770169 PMC3090260

[56] Anzueto A Frutos-Vivar F Esteban A Bensalami N Marks D Raymondos K. Influence of Body Mass Index on Outcome of the Mechanically Ventilated Patients. Thorax (2011) 66(1):66–73. doi: 10.1136/thx.2010.145086 20980246

[57] Ni YN Luo J Yu H Wang YW Hu YH Liu D. Can Body Mass Index Predict Clinical Outcomes for Patients With Acute Lung Injury/Acute Respiratory Distress Syndrome? A Meta-Analysis. Crit Care (2017) 21(1):36. doi: 10.1186/s13054-017-1615-3 28222804 PMC5320793

[58] Maia LA Cruz FF de Oliveira MV Samary CS Fernandes MVS Trivelin SAA. Effects of Obesity on Pulmonary Inflammation and Remodeling in Experimental Moderate Acute Lung Injury. Front Immunol (2019) 10:1215. doi: 10.3389/fimmu.2019.01215 31275296 PMC6593291

[59] Kordonowy LL Burg E Lenox CC Gauthier LM Petty JM Antkowiak M. Obesity is Associated With Neutrophil Dysfunction and Attenuation of Murine Acute Lung Injury. Am J Respir Cell Mol Biol (2012) 47(1):120–7. doi: 10.1165/rcmb.2011-0334OC PMC3402797 22427537

[60] Stapleton RD Dixon AE Parsons PE Ware LB Suratt BT Network NARDS. The Association Between BMI and Plasma Cytokine Levels in Patients With Acute Lung Injury. Chest (2010) 138(3):568–77. doi: 10.1378/chest.10-0014 PMC2940070 20435656

[61] Klionsky DJ Abdelmohsen K Abe A Abedin MJ Abeliovich H Acevedo Arozena A. Guidelines for the Use and Interpretation of Assays for Monitoring Autophagy (3rd Edition). Autophagy (2016) 12(1):1–222. doi: 10.1080/15548627.2015.1100356 26799652 PMC4835977

[62] Finlay S Bray B Lewington AJ Hunter-Rowe CT Banerjee A Atkinson JM. Identification of Risk Factors Associated With Acute Kidney Injury in Patients Admitted to Acute Medical Units. Clin Med (Lond) (2013) 13(3):233–8. doi: 10.7861/clinmedicine.13-3-233 PMC5922664 23760694

[63] Chao CT Wang J Wu HY Huang JW Chien KL. Age Modifies the Risk Factor Profiles for Acute Kidney Injury Among Recently Diagnosed Type 2 Diabetic Patients: A Population-Based Study. Geroscience (2018) 40(2):201–17. doi: 10.1007/s11357-018-0013-3 PMC5964062 29488059

[64] Chang AR Grams ME Ballew SH Bilo H Correa A Evans M. Adiposity and Risk of Decline in Glomerular Filtration Rate: Meta-Analysis of Individual Participant Data in a Global Consortium. BMJ (2019) 364:k5301. doi: 10.1136/bmj.k5301 30630856 PMC6481269

[65] Hsu CY McCulloch CE Iribarren C Darbinian J Go AS. Body Mass Index and Risk for End-Stage Renal Disease. Ann Intern Med (2006) 144(1):21–8. doi: 10.7326/0003-4819-144-1-200601030-00006 16389251

[66] Sarafidis PA. Obesity, Insulin Resistance and Kidney Disease Risk: Insights Into the Relationship. Curr Opin Nephrol Hypertens (2008) 17(5):450–6. doi: 10.1097/MNH.0b013e328305b994 18695384

[67] Rhee CM Ahmadi SF Kalantar-Zadeh K. The Dual Roles of Obesity in Chronic Kidney Disease: A Review of the Current Literature. Curr Opin Nephrol Hypertens (2016) 25(3):208–16. doi: 10.1097/MNH.0000000000000212 PMC5926196 26999023

[68] Kovesdy CP Furth SL Zoccali C World Kidney Day Steering Committee. Obesity and Kidney Disease: Hidden Consequences of the Epidemic. Kidney Int (2017) 91(2):260–2. doi: 10.1016/j.kint.2016.10.019 28010887

[69] Plataki M Kashani K Cabello-Garza J Maldonado F Kashyap R Kor DJ. Predictors of Acute Kidney Injury in Septic Shock Patients: An Observational Cohort Study. Clin J Am Soc Nephrol (2011) 6(7):1744–51. doi: 10.2215/CJN.05480610 21734090

[70] Druml W Metnitz B Schaden E Bauer P Metnitz PG. Impact of Body Mass on Incidence and Prognosis of Acute Kidney Injury Requiring Renal Replacement Therapy. Intensive Care Med (2010) 36(7):1221–8. doi: 10.1007/s00134-010-1844-2 20232041

[71] Friedman AN Guirguis J Kapoor R Gupta S Leaf DE Timsina LR. Obesity, Inflammatory and Thrombotic Markers, and Major Clinical Outcomes in Critically Ill Patients With COVID-19 in the US. Obesity (Silver Spring) (2021) 29(10):1719–30. doi: 10.1002/oby.23245 34109768

[72] Sze S Pan D Nevill CR Gray LJ Martin CA Nazareth J. Ethnicity and Clinical Outcomes in COVID-19: A Systematic Review and Meta-Analysis. EClinicalMedicine (2020) 29:100630. doi: 10.1016/j.eclinm.2020.100630 33200120 PMC7658622

[73] Hawkins RB Charles EJ Mehaffey JH. Socio-Economic Status and COVID-19-Related Cases and Fatalities. Public Health (2020) 189:129–34. doi: 10.1016/j.puhe.2020.09.016 PMC7568122 33227595

[74] Peters EJ Collard D Van Assen S Beudel M Bomers MK Buijs J. Outcomes of Persons With Coronavirus Disease 2019 in Hospitals With and Without Standard Treatment With (Hydroxy)Chloroquine. Clin Microbiol Infect (2020) 27(2):264–8. doi: 10.1016/j.cmi.2020.10.004 PMC7554450 33068758

[75] WHO Solidarity Trial Consortium. Repurposed Antiviral Drugs for Covid-19 - Interim WHO Solidarity Trial Results. N Engl J Med (2020) 384(6):497–511. doi: 10.1056/NEJMoa2023184 33264556 PMC7727327

