# Supplementary material for: Overweight and Obesity Are Associated With Acute Kidney Injury and Acute Respiratory Distress Syndrome, but Not With Increased Mortality in Hospitalized COVID-19 Patients: A Retrospective Cohort Study
Source: Front Endocrinol (Lausanne). 2021 Dec 14;12:747732. doi: 10.3389/fendo.2021.747732 (PMC8713548; doi:10.3389/fendo.2021.747732)
Supplement: Supplementary file 1 [file DataSheet_1.docx]

**Supplemental Material *S1*. Definitions of comorbidities used during data collection**

| Comorbidity | Definition |
| --- | --- |
| AIDS / HIV | Infection with HIV or AIDS |
| Asthma | Physician-diagnosed asthma |
| Autoimmune and/or inflammatory diseases | Autoimmune and/or inflammatory diseases |
| Chronic cardiac disease | Chronic cardiac disease, including congenital heart disease (excluding hypertension) |
| Chronic hematologic disease | Chronic hematologic disease |
| Chronic kidney disease | Chronic kidney disease |
| Chronic neurological disorder | Chronic neurological or degenerative disorders, including (but not limited to) multiple sclerosis, Parkinson’s disease, status after a cerebral vascular event (excluding pain after hernia) |
| Chronic pulmonary disease | Chronic pulmonary disease (excluding asthma) |
| Dementia | Dementia |
| Diabetes with complications | Diabetes mellitus with diabetic retinopathy, nephropathy, or neuropathy |
| Diabetes without complications | Diabetes mellitus without diabetic complications |
| Malignant neoplasm | *Current* malignant neoplasm, including solid neoplasms and acute hematologic malignancies (excluding chronic hematologic malignancies such as chronic lymphocytic leukemia) |
| Mild liver disease | Liver disease without cirrhosis |
| Moderate or severe liver disease | Liver disease with cirrhosis |
| Organ transplant recipient | Organ transplant recipient |
| Rheumatologic disorder | Rheumatologic disorders |

**Supplemental Material *S2*. Evaluation of bias due to missing BMI data**

There were no data missing regarding age. The age was higher in the group without available BMI data (70.1 [58.1-79.0] vs 67.1 [57.0-77.0]). Sex distribution was similar amongst the group with and without available BMI data. However, the availability of the number of pre-existing conditions did differ between groups. Here, the group with missing BMI data more often had a missing number of pre-existing conditions compared to the group with available BMI data (33 vs 0), without differences in sex (p=.918). The age distribution differed significantly, where the group with a missing number of pre-existing conditions was older (75.1 [61.1-84.0] v. 68.0 [57.1-77.1]). In addition, patients with missing BMI data had a shorter length of hospital stay (12.29 [11.27-13.31] v. 16.65 [15.86-17.45]), without significant differences in age or sex.

**Supplemental Material *S3a*. Missing symptoms at admission**

|  | Normal weight  (n=473) | Overweight  (n=669) | Obese  (n=492) | Total  (n=1634) |
| --- | --- | --- | --- | --- |
| Anosmia | 143 (30.2) | 190 (28.4) | 110 (22.4) | 443 (27.1) |
| Use of auxiliary breathing muscles | 133 (28.1) | 185 (27.7) | 115 (23.4) | 433 (26.5) |
| Arthralgia | 119 (25.2) | 158 (23.6) | 95 (19.3) | 372 (22.8) |
| Sore throat | 117 (24.7) | 160 (23.9) | 87 (17.7) | 364 (22.3) |
| Rhinorrhea | 114 (24.1) | 163 (24.4) | 81 (16.5) | 358 (21.9) |
| Myalgia | 109 (23.0) | 148 (22.1) | 90 (18.3) | 347 (21.2) |
| Headache | 107 (22.6) | 138 (20.6) | 77 (15.7) | 322 (19.7) |
| Wheezing | 89 (18.8) | 136 (20.3) | 72 (14.6) | 297 (18.2) |
| Hemoptysis | 64 (13.5) | 95 (14.2) | 52 (10.6) | 211 (12.9) |
| Cough | 66 (14.0) | 83 (12.4) | 57 (11.6) | 206 (12.6) |
| Confusion | 61 (12.9) | 91 (13.6) | 49 (10.0) | 201 (12.3) |
| Nausea and/or vomiting | 69 (14.6) | 83 (12.4) | 47 (9.6) | 199 (12.2) |
| Abdominal pain | 65 (13.7) | 86 (12.9) | 43 (8.7) | 194 (11.9) |
| Chest pain | 67 (14.2) | 85 (12.7) | 42 (8.5) | 194 (11.9) |
| Diarrhea | 60 (12.7) | 79 (11.8) | 36 (7.3) | 175 (10.7) |
| Fever | 41 (8.7) | 63 (9.4) | 30 (6.1) | 134 (8.2) |
| Fatigue/malaise | 41 (8.7) | 49 (7.3) | 32 (6.5) | 122 (7.5) |
| Dyspnea | 27 (5.7) | 32 (4.8) | 19 (3.9) | 78 (4.8) |

Description of missing symptoms at admission presented as count (%) per group and total.

**Supplemental Material *S3b*. Missing laboratory findings at admission**

|  | Normal weight  (n=473) | Overweight  (n=669) | Obese  (n=492) | Total  (n=1634) |
| --- | --- | --- | --- | --- |
| Total bilirubin, µmol/L | 179 (37.8) | 255 (38.1) | 161 (32.7) | 595 (36.4) |
| Aspartate aminotransferase, U/L | 164 (34.7) | 227 (33.9) | 146 (29.7) | 537 (32.9) |
| Lactate dehydrogenase, IU/L | 157 (33.2) | 224 (33.5) | 149 (30.3) | 530 (32.4) |
| Glucose, mmol/L | 156 (33.0) | 217 (32.4) | 127 (25.8) | 500 (30.6) |
| Alanine aminotransferase, U/L | 142 (30.0) | 183 (27.4) | 119 (242) | 444 (27.2) |
| C-reactive protein, mg/L | 114 (24.1) | 154 (23.0) | 94 (19.1) | 362 (22.2) |
| Platelets, X10^9^/L | 113 (23.9) | 146 (21.8) | 89 (18.1) | 348 (21.3) |
| Creatinine, µmol/L | 102 (21.6) | 141 (21.1) | 86 (17.5) | 329 (20.1) |
| Hemoglobin, mmol/L | 104 (22.0) | 138 (20.6) | 86 (17.5) | 328 (20.1) |
| White blood count, × 10^9^/L | 104 (22.0) | 137 (20.5) | 85 (17.3) | 326 (20.0) |

Description of missing laboratory data at admission presented as count (%) per group and total.

**Supplemental Material *S3c*. Missing complications during admission**

Information on complications during admission was missing for 10.9-18.7% of the variables.

|  | Normal weight  (n=473) | Overweight  (n=669) | Obese  (n=492) | Total  **(n=1634)** |
| --- | --- | --- | --- | --- |
| Aspergillosis pneumonia | 94 (19.9) | 136 (20.3) | 76 (15.4) | 306 (18.7) |
| Anemia | 52 (11.0) | 89 (13.3) | 42 (8.5) | 183 (11.2) |
| Bacteremia | 51 (10.8) | 89 (13.3) | 42 (8.5) | 182 (11.1) |
| ARDS | 51 (10.8) | 90 (13.5) | 41 (8.3) | 182 (11.1) |
| Congestive heart failure | 52 (11.0) | 89 (13.3) | 41 (8.3) | 182 (11.1) |
| Endocarditis/Myocarditis/Pericarditis | 53 (11.2) | 87 (13.0) | 41 (8.3) | 181 (11.1) |
| Gastrointestinal hemorrhage | 51 (10.8) | 89 (13.3) | 41 (8.3) | 181 (11.1) |
| Liver failure | 52 (11.0) | 89 (13.3) | 41 (8.3) | 182 (11.1) |
| Rhabdomyolysis or Myositis | 52 (11.0) | 89 (13.3) | 41 (8.3) | 182 (11.1) |
| Cerebrovascular accident | 52 (11.0) | 87 (13.0) | 41 (8.3) | 180 (11.0) |
| Coagulation disorder | 51 (10.8) | 88 (13.2) | 41 (8.3) | 180 (11.0) |
| Delirium | 49 (10.4) | 88 (13.2) | 43 (8.7) | 180 (11.0) |
| Pneumothorax | 51 (10.8) | 88 (13.2) | 41 (8.3) | 180 (11.0) |
| Seizure | 52 (11.0) | 87 (13.0) | 41 (8.3) | 180 (11.0) |
| Bacterial pneumonia | 50 (10.6) | 88 (13.2) | 41 (8.3) | 179 (11.0) |
| Cardiac arrhythmia | 50 (10.6) | 88 (13.2) | 41 (8.3) | 179 (11.0) |
| Cardiac ischemia | 52 (11.0) | 88 (13.2) | 41 (8.3) | 179 (11.0) |
| Acute renal failure/injury requiring dialysis | 50 (10.6) | 87 (13.0) | 41 (8.3) | 178 (10.9) |

Missing data on complications during hospital admission. Data is presented per complication as count (%) per group and total.

**Supplemental Table *S4*. Post-hoc analysis of demographic and baseline characteristics of hospitalized patients with COVID-19**

|  | Normal weight vs overweight | Normal weight vs obesity | Overweight vs obesity |
| --- | --- | --- | --- |
| Male sex (%) | 0.048 | 0.012 | < 0.001 |
| Age (years) | < 0.001 | < 0.001 | 0.001 |
| Hypertension (%) | 0.069 | < 0.001 | 0.006 |
| Diabetes, with complications (%) | 0.450 | 0.042 | 0.663 |
| Diabetes, without complications (%) | 1.000 | < 0.001 | 0.003 |
| Organ transplant | 0.162 | 0.066 | 1.000 |
| Asthma (%) | 0.336 | 0.018 | 0.522 |
| Hematologic disease (%) | 0.393 | 0.015 | 0.381 |
| Malignancy (%) | 0.012 | < 0.001 | 0.927 |

Post-hoc pairwise analysis of significantly different demographic characteristics between patients with normal weight (BMI 18.5-24.9 kg/m^2^), overweight (BMI 25-29.9 kg/m^2^) and obesity BMI (≥ 30 kg/m^2^). P-values have been adjusted by the Bonferroni correction for multiple testing.

**Supplemental Table *S5*. Medication use prior to hospital admission**

|  | Normal weight  (n=473) | Overweight  (n=669) | Obese  (n=492) | *p-value* |
| --- | --- | --- | --- | --- |
| Home medication use, yes | 401 (85.1) | 552 (83.1) | 428 (87.5) | 0.118 |
| Antiplatelet drugs | 45 (9.5) | 62 9.3) | 42 (8.5) | 0.857 |
| Coumarin derivatives | 17 (3.6) | 19 (2.8) | 13 (2.6) | 0.654 |
| Direct oral anticoagulants | 13 (2.7) | 22 (3.3) | 20 (4.1) | 0.520 |
| Lipid lowering medication | 59 (12.5) | 107 (16.0) | 76 (15.4) | 0.229 |
| Oral glucose lowering medication | 24 (5.1) | 54 (8.1) | 46 (9.3) | 0.036 |
| Insulin | 17 (3.6) | 20 (3.0) | 28 (5.7) | 0.059 |
| Antiarrhythmic drugs | 7 (1.5) | 6 (0.9) | 7 (1.4) | 0.604 |
| Beta blocker | 49 (10.4) | 72 (10.8) | 64 (13.0) | 0.361 |
| Digoxin | 4 (0.8) | 5 (0.7) | 4 (0.8) | 1.000 |
| Diuretics | 41 (8.7) | 63 (9.4) | 64 (13.0) | 0.054 |
| ACE inhibitor | 26 (5.5) | 45 (8.2) | 24 (6.9) | 0.382 |
| Angiotensin II receptor blocker | 19 (4.0) | 55 (8.2) | 34 (6.9) | 0.018 |
| Aldosterone antagonist | 2 (0.4) | 7 (1.0) | 3 (0.6) | 0.508 |
| Calcium channel blocker | 21 (4.4) | 51 (7.6) | 30 (6.1) | 0.090 |
| Other cardiovascular medication | 5 (1.1) | 9 (1.3) | 9 (1.8) | 0.586 |
| Immune suppressive medication | 46 (12.0) | 43 (7.9) | 37 (8.8) | 0.095 |

Data are count (%) and compared by chi-square test or Fisher’s exact test.

**Supplemental Table *S6*. Post-hoc analysis of symptoms at admission**

|  | Normal weight vs overweight | Normal weight vs obesity | Overweight vs obesity |
| --- | --- | --- | --- |
| Dyspnea | < 0.001 | < 0.001 | 0.600 |
| Coughing | 0.009 | 0.078 | 1.000 |
| Headache | 0.090 | < 0.001 | 0.255 |
| Chest pain | 0.021 | 0.003 | 1.000 |

Post-hoc pairwise analysis of significantly different presenting symptoms during admission between patients with normal weight (BMI 18.5-24.9 kg/m^2^), overweight (BMI 25-29.9 kg/m^2^) and obesity BMI (≥ 30 kg/m^2^). P-values have been adjusted by the Bonferroni correction for multiple testing.

**Supplemental Table** ***S7a*. Laboratory findings at admission**

|  | Normal weight | Overweight | Obese | *p*-value |
| --- | --- | --- | --- | --- |
| Hemoglobin, mmol/L | 8.0 [7.0-8.7] | 8.5 [7.7-9.1] | 8.4 [7.6-9.2] | < 0.001 |
| White blood count, × 10^9^/L | 7.3 [5.2-9.8] | 6.9 [5.2-9.5] | 6.8 [5.1-9.1] | 0.535 |
| Platelets, × 10^9^/L | 221 [169-294] | 215 [168-265] | 208 [168-270] | 0.223 |
| Creatinine, µmol/L | 82 [65-114] | 87 [70-113] | 85 [69-111] | 0.250 |
| Alanine aminotransferase, U/L | 30 [20-52] | 35 [24-54] | 36 [24-52] | 0.007 |
| Aspartate aminotransferase, U/L | 44 [32-66] | 46 [34-70] | 48 [33-65] | 0.464 |
| Total bilirubin, µmol/L | 8.4 [6.0-12.7] | 9.0 [6.0-12.3] | 8.0 [6.0-12.0] | 0.141 |
| Glucose, mmol/L | 6.8 [5.9-8.0] | 6.9 [6.1-8.6] | 7.3 [6.2-9.8] | < 0.001 |
| C-reactive protein, mg/L | 89 [46-147] | 93 [46-158] | 84 [45-154] | 0.439 |
| Lactate dehydrogenase, IU/L | 329 [246-441] | 356 [281-473] | 346 [262-464] | 0.044 |

Laboratory findings at admission showed a significant difference in hemoglobin, alanine aminotransferase, glucose and lactate dehydrogenase between groups. Data are median [IQR] and compared by Kruskal-Wallis test.

**Supplemental Table** ***S7b*. Post-hoc analysis of significant laboratory findings**

|  | Normal weight vs overweight | Normal weight vs obesity | Overweight vs obesity |
| --- | --- | --- | --- |
| Hemoglobin, mmol/L | <0.001 | <0.001 | 1.000 |
| Alanine aminotransferase, U/L | 0.020 | 0.012 | 1.000 |
| Glucose, mmol/L | 0.203 | 0.000 | 0.013 |
| Lactate dehydrogenase, IU/L | 0.038 | 0.392 | 1.000 |

Post-hoc pairwise analysis of significant laboratory findings. P-values have been adjusted by the Bonferroni correction for multiple testing.

**Supplemental Table *S8.* Complications during admission**

|  | Normal weight  (n=473) | Overweight  (n=669) | Obese  (n=492) | Total  **(n=1634)** |
| --- | --- | --- | --- | --- |
| **Pulmonary complications** |  |  |  |  |
| ARDS | 86 (20.4) | 178 (30.7) | 107 (23.7) | 371 (25.6) |
| Bacterial pneumonia | 46 (10.9) | 81 (13.9) | 49 (10.9) | 176 (12.1) |
| Aspergillosis pneumonia | 8 (2.1) | 12 (2.3) | 11 (2.6) | 31 (2.3) |
| Pneumothorax | 13 (3.1) | 14 (2.4) | 5 (1.1) | 32 (2.2) |
| **Cardiac complications** |  |  |  |  |
| Congestive heart failure | 22 (5.2) | 14 (2.4) | 20 (4.4) | 56 (3.9) |
| Endocarditis/myocarditis/ pericarditis | 4 (1.0) | 4 (0.7) | 2 (0.4) | 10 (0.7) |
| Cardiac arrhythmia | 46 (10.9) | 66 (11.4) | 37 (8.2) | 149 (10.2) |
| Cardiac ischemia | 6 (1.4) | 10 (1.7) | 5 (1.1) | 21 (1.4) |
| **Neurologic complications** |  |  |  |  |
| Seizure | 5 (1.2) | 6 (1.0) | 2 (0.4) | 13 (0.9) |
| Cerebrovascular accident | 8 (1.9) | 15 (2.6) | 9 (2.0) | 32 (2.2) |
| **Coagulation disorders** |  |  |  |  |
| Pulmonary embolism | 30 (6.3) | 52 (7.8) | 28 (5.7) | 110 (6.7) |
| Deep venous thrombosis | 13 (2.7) | 22 (3.3) | 8 (1.6) | 43 (2.6) |
| Disseminated intravascular coagulation | 3 (0.6) | 5 (0.7) | 1 (0.2) | 9 (0.6) |
| **Other complications** |  |  |  |  |
| Bacteremia | 33 (7.8) | 62 (10.7) | 40 (8.9) | 135 (9.3) |
| Anemia requiring transfusion | 41 (9.7) | 58 (10.0) | 32 (7.1) | 131 (9.0) |
| Rhabdomyolysis or Myositis | 8 (1.9) | 14 (2.4) | 20 (4.4) | 42 (2.9) |
| Acute renal failure/injury requiring dialysis | 19 (4.5) | 57 (9.8) | 34 (7.5) | 110 (7.6) |
| Gastrointestinal hemorrhage | 3 (0.7) | 11 (1.9) | 6 (1.3) | 20 (1.4) |
| Liver failure | 8 (1.9) | 10 (1.7) | 5 (1.1) | 23 (1.6) |
| Delirium | 73 (17.2) | 114 (19.6) | 73 (16.3) | 260 (17.9) |
| Other complications | 48 (11.3) | 82 (14.1) | 60 (13.3) | 190 (13.1) |

Descriptive data on complications during admission. Data are count (%) and presented per group and overall.

**Supplemental Table *S9*.** **Duration of hospital stay, ICU stay, and mechanical ventilation**

|  | Normal weight | Overweight | Obese | *p-value* |
| --- | --- | --- | --- | --- |
| Hospital stay  N | 9.0  [4.0-24.0]  467 | 11.0  [4.0-30.0]  659 | 9.0  [4.0-25.0]  485 | 0.059 |
| ICU admission  N | 9.0  [6.0-15.0]  104 | 12.0  [5.0-24.0]  223 | 12.0  [5.5-19.0]  140 | 0.088 |
| Ventilation duration  N | 8.0  [5.0-14.5]  87 | 12.0  [6.0-22.0]^a^  190 | 11.0  [7.0-17.5]  117 | 0.002 |

Data are median [IQR] and compared by Kruskal-Wallis and post-hoc Mann-Whitney-U test. N indicates the number per group. ^a^ p=<0.001 vs the normal weight group.
